# Virginia State Dental Association—Sixth Annual Session

**Published:** 1876-03

**Authors:** 


AMERICAN JOURNAL

OF

DENTAL SCIENCE.

Vol. IX. THIRD SERIES—MARCH, 1876. No. 11.

### ARTICLE I.

### *Virginia State Dental Association—Sixth Annual Session.*

(*Reported Expressly for this Journal.*)

THIRD DAY.—*Morning Session.*

The Association met pursuant to adjournment, the President in the chair.

The report of the committee on Mechanical Dentistry was called for. It was stated that none of the committee were present.

The subject of the use of Celluloid having been broached, Dr. J. F. Thompson, of Fredericksburg, gave his views concerning this base. He had used it for three years to the exclusion of rubber, and with such satisfactory results as to induce him to continue its use permanently. He hoped that the members present who had used or experimented even with celluloid, would report their views, giving not only their successes with it, but their failures also. For his part he had made not a single failure so far. Compared with rubber he thought it far superior; his patients greatly preferred it, in fact refused to have rubber. He had no hobby to ride, was only desirous of getting the best results for his patients, and he was sincere in his conviction of its superiority to vulcanite.

Dr. Henkel, of Staunton, had used celluloid for over three years. Had made twenty-two pieces, had replaced four of these. His experience was that it changed color in the mouth. He thought the plates lately made better than the first ones. Had learned from his failures something of the peculiarities of celluloid; these required to be consulted and overcome. If it proved as satisfactory in the future as in the past, he would not use vulcanite again. It flows better than the latter, was sufficiently strong, and of a good color.

Dr. Frank Harris, of Harrisonburg, said he had commenced the use of celluloid in February last. He had worked seven plates; of these, two only were still in wear. One of the plates he inserted was of so poor a character that it warped from the contact of hot tea on the first trial. The lady wore it home took hot tea for her supper, and found the plate warped out of all shape. He could not think it equal to vulcanite.

Dr. Thompson thought it ought to be kept in water when not in the mouth. He had not seen any warping in his own practice, and had met with no such failures as the last speaker reported.

Dr. Keesee, of Richmond, thought that if we would give celluloid a little time, the failures would be less numerous. It certainly seemed very strong; the tests he had applied had proved it superior to vulcanite in this respect. The question of its permanency was of course only to be settled by time.

Dr. Johnston, of Staunton, reported having seen a piece in use over four years in which no change was apparent. There was neither discoloration, flaking or warping. All the pieces he had made were satisfactory, and he preferred it to any plastic base he had seen.

Dr. Thackston, (Dr. Moore in the chair.) I have been in the profession for many years. I entered it when no such thing as plastic work was known. I have never been a hobby-rider, but aimed at conservatism. I commenced with gold, when most pieces were made of that material. I consider it now, as then, the perfection of bases for dental plates. Platinum, palladium, silver, in turn had been tried; and these were succeeded by cheoplastic work, and this again by rubber. I am familiar with the whole history of the vulcanite question, and can assure you gentlemen, that the difficulties that are now attending the introduction of celluloid, and its adaptation to dental uses, are not to be compared with those that accompanied the use of rubber at its inception. With regard to vulcanite I cannot think it in any way comparable to celluloid. Its color is bad, it acts as a local irritant, it is indeed in many cases a positive poison. I have no prejudices to foster. While I used the rubber, I cheerfully paid the royalty so long as the Goodyear patent was in existence, as I considered it a valid and just claim. When it expired and the Goodyear Dental Vulcanite claimed royalty on the Cummings' patent I declined to pay it, deeming it an unjust and wicked tax. I did not, however, in a covert way seek to avoid payment of this tax, and at the same time secretly endeavor to continue the use of vulcanite, but at once laid it aside, discontinuing its use entirely. For a time I was without any satisfactory plastic base as a substitute for rubber, and for the cases in which I had demand for a cheap base I used silver or platinum. Three or four years ago my attention was called to celluloid, then first being introduced. The first pieee I made was moulded after the old oil process, with gum teeth. That piece now over three years old, is still giving satisfaction, and is perfectly successful. I have found celluloid more available and more satisfactory than rubber. I would not return to the use of rubber if Bacon & Co. would offer me a fee simple right to use without cost forever. I have wholly, utterly and finally discarded and rejected vulcanite as a base for dental plates. I think, as has been suggested, that the plates should be well seasoned, and time taken in their moulding.

Dr. Keesee. Can Dr. Thackston give a satisfactory explanation of the dark line we see around or near the vacuum cavity in celluloid plates?

Dr. Thackston. I have seen no explanation that is satisfactory,—the theory advanced by a writer in the *Dental Cosmos*, that it was due to a lapping of the plate at that point, I hardly think fully accounts for it.

Dr. Frank Harris thought that rubber had greatly improved—the whalebone and bowspring varieties being greatly superior to the red rubber of 1868-70. With regard to celluloid his failures were too manifest and palpable to be counted as accidental. He hoped that the day would come when rubber could be discarded, but at present he confessed he saw but little hopes of relief in celluloid as a substitute. He had never seen a case of rubber disease, so called.

Dr. Cowardin had seen two cases of rubber poisoning cured by the insertion of metallic plates.

Dr. Henkel was sure that rubber plates did produce poisonous results.

Dr. Perrow had never seen a case of mercurial poisoning from a rubber plate. He could not see how celluloid could take the place of rubber, if the testimony of those who had spoken here was to be accepted.

Dr. Sydnor, of Salema, Va., thought that perhaps the failure in the case of Dr. Harris, might have been due to the imperfect seasoning of the plates. He inquired if it was known that celluloid would answer for regulating plates where thinness and elasticity were required.

Dr. J. Hall Moore, of Richmond, said that it was well suited to just this class of work. His opinion of its strength and firmness was corroborated by Drs. Keesee and C. A. Mercer, of Richmond.

Dr. Thackston. We should not condemn celluloid because of a few failures. It is unreasonable to expect perfect success with anything, especially with so new and only partially developed a substance as this. We should consider the great number of failures that had been made in continuous gum, how many years of failuse preceded success.

Dr. Frank Harris replied defending his position. He could not attribute the failure he had made to faulty manipulations. No one would rejoice more than he to know of a good substitute for rubber, but he was afraid to take celluloid as that substitute.

Prof. Hodgkin. Celluloid has its peculiarities which must be recognized and complied with before it can be successfully worked. It is yet in its infancy. But depend upon it the time must come in its history, that this infant will go forth strong and in every way desirable. It has been well said here this evening, that we should not condemn it hastily for a few faults, nor cast it aside because we do not at first accomplish all with it that we could hope or desire. It is certain that in beauty, naturalness of appearance and the perfection with which it copies the finest lines, it is superior to any plastic base so far produced. Its durability can only be tested by time, but if the testimony of not only the larger majority of those here present, but of dentists all over the land can be believed, it is a satisfactory substitute for rubber in all those qualities which render that base desirable, and is vastly superior to it in color and exemption from the suspicion of mercurial poisoning. The dry heat is better than oil or steam, and if the pieces are reinvested after polishing and kept at a moderate temperature, (say 120° or 130°) for eight or ten hours, much of the camphor is driven off. When it is desirable to slightly change the color of the gum, the etheral solution known as collodion, (that which is colored pink,) may be painted on the gum and will change the tone. A thermometer is simply in the way.

The subject was further discussed, and the subject of mechanical dentistry was passed.

Dental Pathology was called. An essay from the pen of Dr. Burkholder, of Harrisonburg, Va., "On the Preservation of the Deciduous Teeth, was read.

Adjourned.

*Afternoon Session.*

The Association re-assembled at 3 P. M.

The subject of Operative Dentistry was called.

Dr. Jeter, of Richmond, was not prepared with a report, he was not the chairman of the committee.

Dr. Thackston. It is to be hoped that in future the members of the various committees will not rely on their respective chairmen to prepare reports, but that each member will look on his appointment as an indication that he is expected to present a report independent of any that may be made by the chairman. We should not look to the chairman and conclude that because he is the head of the committee, that therefore he is to do the work of the committee. Let each member while co-operating with the others, yet not rely on them to do his work.

Dr. Jeter. I hope sir, that the Association may have the benefit of the counsel of Prof. Hodgkin, who, coming from Baltimore, the headquarters of Dentistry, should be well posted.

Prof. Hodgkin. I fear Mr. President, that I shall lay myself open to the imputation of being old-fashioned, in prefering and adhering to old methods and old instruments, to old forms of foil and old styles of work. The longer I live and practice dentistry, the more strongly I feel the difficulties of the position I occupy, both as a teacher and as a practitioner. When I hear gentlemen say, as I do sometimes, that they have no trouble in performing this or that operation, that they never fail in their manipulation of this or that form of gold—usually the newest form,—and when no failures are reported in celluloid or other styles of work, I am inclined to feel envious of them, and to lament my own failures and shortcomings. It has seemed to me that now-a-days one is thought not to be up with the times unless he uses the thickest and most adhesive of gold foil which must be driven into the cavity and against its frail walls, with mallets of ever so many muscle power. That unless the operator has a burring engine, and a pneumatic, automatic, hydraulic, electric or some other motive-power mallet; that unless he is equipped with rubber dam and clamps and all the multitudinous apparatus of the present day, that his mission will be a failure, and his practice ooze away into the hands of the more fortunate possessor of these nice things. But, Mr. President, I cannot ignore the fact that long before the days of mallets and engines, of double action engines and rubber dam, work was done, that was in every respect, in so far as service to the patient was concerned, and durability, and of æsthetic beauty, equal to anything that is now-a-days produced. I do not involve in a wholesale condemnation all these improved adjuncts—they are most useful and important in place, but the fear is that our young men will be too much inclined to depend on these to the exclusion of the pains-taking care and thoughtful manipulation dentists formerly gave their work. All these things are valuable, but they are not dentistry; they are not and cannot be a substitute for honest toil and painstaking. I confess my attachment to the old fashioned soft foil and hand pressure. I have tried and found wanting many of the methods vaunted as the "best thing out," best apparently, because newest; and I consider that for the most part, I do better work, get more gold in a cavity with hand pressure and foil of which Abbey's old soft gold foil is the type, than with any of the modern styles. I do not reject adhesive foil, but use it as the exception and not the rule, and depend greatly on a lubricant known as "elbow grease."

Dr. J. Hall Moore, of Richmond. I think the profession is drifting back to old practices and old methods. I have laid aside for some time my automatic mallet, and dropped the use of adhesive gold. I use non-adhesive gold in strips or cylinders. I have no faith in sponge gold, the numerous failures in my practice and seen in the practice of others, have led me to abandon its use. I shall be considered by some I fear, as an "old fogy," but I have been so long in the way of using plain foil that I do not feel like changing to anything else, unless the advantage of doing so is shown to be unquestionable.

Dr. Moore here exhibited a small brush to be used with the burring engine for polishing teeth and fillings, which he found very useful.

Prof. Hodgkin. Let us understand what we mean by soft foil. I think the confusion and obscurity on this subject is considerable, and due to a want of clear application of the properties of gold. It has been well said by Dr. Latimer, of New York, that gold, be it foil or plate, is softened by annealing. Why then is it that we find the plate is more easily swaged after annealing, and the foil is less easily manipulated? It is not that the foil is made harder, though it seems to be so, but that it has lost its adjustability. A piece of gold is placed in position in the cavity, and if of unannealed gold, the succeeding piece is easily pushed against or past it into position. Its adjustability is at the maximum. But if it be adhesive, (or cohesive if we prefer that term,) it sticks fast one piece to the other,—its cohesion being in the way of its adjustability, this last quality is at its minimum. Evidently, a quality in foil which enables us to place it in any position we desire, which is not in the way of its going nearer to its place is eminently desirable; a foil which does not possess this trait, whatever value we may attach to its cohesive properties,—and that this property is valuable for some purposes, I cheerfully admit,—cannot be so generally useful as the soft, or plain, or adjustable foil.

Dr. W. Leigh Burton, of Richmond, stated that he used soft gold foil in filling teeth, generally using as fine as No. 4.

His manner of preparing it as follows: He takes a sheet and cuts it into three strips of equal width, and *ropes*

them. Each rope is then cut into sections more or less, according to the size of the cavity to be filled, and each section folded in the form of a pellet. In case of a very large cavity, the whole or half of a sheet may be used, prepared in the same manner.

He uses a fine pointed plugger with a cut across the point, or slightly roughened with a file. Each pellet is taken on the point of the plugger, placed in its proper position in the cavity, *taking care a'ways to allow a surplus of gold to project above the margin of it.* In large cavities, after the pellet has been placed in position, a smooth faced foot-shaped plugger, and a few blows from a mallet, will assist greatly in giving solidity to the filling; and thus the operation is continued until the cavity has received all the gold it will hold. The next operation is that of consolidation, and in this the use of the surplus gold around the margin of the cavity will be made manifest. Still using a fine pointed instrument, this is consolidated at first by hand pressure, and completed by a stout fine pointed instrument and mallet. In the use of the mallet for this purpose, great dexterity may be attained, with absolutely little or no inconveniences to the patient. Holding the instrument in the left hand, and causing it to spring off a short distance from the filling, the right-hand is enabled to administer the blows with a rapidity equal to if not greater than any automatic instrument. The operation is then completed in the ordinary manner by grinding down the surplus gold, and polishing the filling.

Dr. L. M. Cowardin, of Richmond, used Pack's foil. He thought there was a great difference of foils, even of the same manufacture; thought that the foil which was in the books with smooth paper leaves, worked better than that between rough paper. He had been so struck with this as to lead him to select the books with smooth paper exclusively.

Dr. J. F Thompson, of Fredericksburg, said that in his attendance on the meetings of the Southern Dental Association, from year to year, he was struck with the fact mentioned by Dr. Moore, that the dentists were drifting back to soft foil. He found he could do more with soft foil than with adhesive, and used it almost exclusively. His method was to fill the cavity with unannealed foil, and then add a little of the same foil annealed for finishing the surface. He thought that in this way he secured the best results, the annealed foil adhering strongly to that already in the cavity, and giving a hardness to the surface not attainable by the soft foil alone.

Dr. Burton. I do not use annealed foil for finishing my fillings. I can gain as good a finish to them with the plain foil as I desire.

Prof. Hodgkin. If I may be allowed to criticise the method of Dr. Thompson, and others who have spoken, I would say that I must dissent from their views as here expressed. It is allowed on all hands, by all who have spoken, that soft foil is used in preference to the adhesive, because of its property of adjustability, by reason of which it can be forced against the walls of the cavity more closely; in a word, because better margins to the filling can be made. Now it does seem to me that the position assumed by the gentlemen who advocate this mixed use of foil, is most unfortunate,—for if it be a fact, that soft foil goes closer to the walls, it is a more important fact still, that the margins of the cavity are just where we want the very closest possible adaptation. If it be true that adhesive foil will not make as good borders as soft foil, there is no escape from the conclusion—they have made it for themselves—that the surface, if anywhere is the place for foil of this soft character. If I am to make a combination filling of two varieties of gold foil, then it does seem to me, that anywhere than at the finishing, would be the place for the adhesive foil.

Dr. Frank Harris gave an account of an operation he had performed—the building up of an incisor, in which he had laid the foundation of the filling with Hasting's gold annealed, and made the bulk of the work of Watt's crystal gold. He felt that the operation was one that would be durable, and while he had not, as some, gone crazy on the subject of adhesive foil and building up teeth, yet he was not prepared to accept the views of those who so sweepingly denounced all gold and all work that was not of the strictly non-cohesive type.

Dr. Henkel had used and experimented with a number of varieties of gold, and he felt when he wished to do work that would prove permanent, he preferred soft foil.

Dr. Moore. I would call the attention of members of this Association, to a filling formerly considerably used, but for some time out of fashion. It was my practice in years past, to put in a good many fillings of gold and tin foil combined, and I confess the fillings thus made, have proved more durable than most others. I hardly know why I left off this method, but I feel convinced that it is a most useful and enduring filling, requiring considerably less labor to insert than gold, and possessing a most remarkable property of hardening like amalgam, the whole filling apparently becoming as hard almost as steel. There seemed to be no galvanic action in the two metals thus combined, and it apparently had the effect of hardening the walls of the tooth like amalgam.

The subject was passed, and that of Dental Education was called.

Dr. Burton said the committee had no report. One new book he believed had been published, that of Salter, but he was not advised as to its contents.

The new constitution was read and adopted.

The following essay was read by Prof. Hodgkin.

It is a source of great gratification to those of us who are trying to educate the future dentist to know of the stir that is going on on this subject of dental education. It is a sign of the times prophetic of better things for those who teach and those who are to be taught; and as better for the masses who used the services of the dentist. Earnest men are at work at this problem of education, and if good does not result I shall be mistaken. It is well that this agitation is going on; it is doubtless a sign of something at fault; but as instruction in all things is gradual, it may be but not to hurry too much the changes we may see needed in our present methods of teaching. Sudden and violent changes in any department of study are to be dreaded.

We should take into account the youth of our profession, take into consideration the amazing rapidity which has haracterized its growth. Let its bones solidify and itsc muscular system get tone, before we criticise its numerous organizations.

The colleges are doing well. If it were put to the test we would see that in so far as anatomy, physiology and chemistry are taught, they are taught quite as well if not better than in medical schools.

This subject of dental education is strangely misunderstood by the profession at large.

No doubt there are defective methods of reaching in use, no doubt the curriculum of our colleges is not just what is best, methods of teaching are human, and our work in this direction is as liable to error, to impufaction, to want of success, as any other purely human undertaking.

How easy it is for those outside to say how a college should be conducted.

*The causes of apparent failure.* I have said *apparent*, for it is only such. No real failure I think is seen in the work of our dental colleges that would not more readily occur or to a greater extent in a medical or law school. If we reflect for an instant on the incompetent men who are turned out in droves annually from the various institutions of learning, to crowd the lower stories of the professions, we may feel thankful that we are in as good a condition to throw stones at others as we are. I feel sure that if some "mind oneter" test could be applied to the multitudinous throngs, who, hungry for fees and places issue forth to "locate," it would be found that those coming from dental schools would compare favorably with others.

Dental schools do all for their students, (allowing for the frailty and imperfection of human teaching,) that the material they have to work on will allow.

"You can't make a silk purse, &c." Those who come with fair brains and a fair preliminary training, graduate with credit as a rule. But how many are found for whom no one could ever hope substantial success in anything.

Here then is the root, or the roots of the evil. First, want of capacity. Second, want of adequate preliminary. The first class is comparatively small; training the second is large, much larger than is usually imagined.

At first sight one might suppose that such an element, such as is first mentioned, might readily be eliminated from the classes; it is not so easy. With very many who enter on the study of anything it is only by a process of evolution, often of a slow and painful sort that the gradual development of faculties can be brought out. You cannot ask a new man and in a casual inspection determine his fitness, his qualifications for dentistry. Few will deny the necessity for peculiar gifts, but often these lie dominant and undeveloped until by the slow dawning of light, his faculties become fully apparent, and he stands before you with talents which until then you little dreamed of. I confess my great surprise and astonishment at the development in students of capacity whom I had considered hopeless cases or nearly so. I know that occasionally an almost full-pledged dentist comes along, but how rare these are, any dental professor—notably any demonstrator—will tell you. Your youth who is the pride of his doting parents, who can mend your clock or take your wife's sewing machine to pieces, is not necessarily a dentist even in embryo, and too often his natural gifts in this mechanical direction are sadly in the way of his advancement, as the self-conceit that often give rise to, is an effectual bar to advancement From all students, deliver me from him who knows all about it before hand.

After all, the proportion of those students who enter the dental profession not gifted beforehand with natural ability, either dominant or manifest, is, I think not large. I feel every year as I teach, that the difficulty is so much not in want of natural aptitude—though this is no mean trait—as it is in defective mental culture. Depend upon it gentlemen, the world is getting to realize more and more every day the importance of the doctrine, of the superiority of mind over matter. It is your scientific farmer who beats the practical man; it is science applied every day in all departments of industry that is acheiving results that your practical man lies in wait to sieze and use all the time it may be dividing the work of the toiler without whom he could not subsist. Science is power, dominion, control over matter, and in so far as the dental student masters it, in so far will he be able to make his practice a success.

Defective mental culture is, I feel sure, at the bottom of many men's failures, (assuming that there is mind to cultivate,) and no less of dentists than other people. How few can analyze a proposition, state a fact clearly, reason out a deduction. Indeed there are few of us who feel more matured, who are called by reason of supposed peculiar gifts to fill chairs in colleges, who do our own thinking. How few are the thinkers. We can count them on our finger's ends, and not use up all the digits by any means.

So long as dentists are no more selective in their material for office students than they are, so long will the colleges find trouble in their subsequent work. It is with little discrimination apparently that dentists take private students. I will not say that they are influenced by unworthy motives, but the material they forward to us with letters of recommendation, certainly does not always reflect credit on their judgment.

The colleges ask the support of the profession. But it is well for the profession to understand clearly the character of the support asked for. It is not to crowd the seats of our lecture rooms with crude material, it is not to forward to us under certificate students who are able to make a rubber plate and nothing more. It is not to send us yonng men whose ability is altogether in their fingers, but it is to send us students who are able to intelligently follow a plain lecture on anatomy, etc., who are not obliged to be taught the rudiments of the English language; who are sufficiently posted in elementary physiology, anatomy, chemistry and the special relations of their sciences to dentistry to comprehend technical terms, and a great reform will have been worked.

How much time is spent in teaching students that which they ought to have mastered in school, would astonish any one not conversant with the subject. The amount of ignorance of the simpler, scientific facts is distressing. If it were so in dental schools only, it might be used to prove that the material of the profession is poor; I colld readily show you that it is just as bad in the medical profession.

The cure is in the hands of you who undertake the preliminary training of the student. The colleges can be made by you just what you wish them to be. But when students habitually sit in our lecture rooms and yarn at words and phases unintelligible to them, when students show by their very acts as some do, that their desire is not to get an education so much as to get a diploma, is it any wonder that lectures are dispirited, or that dental education seems to flag.

Dr. J. F. Thompson, read an essay on Irregularity of the Teeth as produced by Thumb Sucking.

Drs. Henkle, Steele and Prof. Hodgkin discussed this paper, both thinking that the habit of thumb sucking was not to any great extent the cause of irregularity.

The subject of voluntary essays was passed.

Prof. Hodgkin. I ask the attention of this Association to a case which presents great interest, and some of the points of which I confess are so startlingly new, as to cause grave doubts as to its proper representation to me by the parties who related it, and yet I have no reason to doubt their veracity. I called last night to see an old friend whom I had not seen for some years, and in the course of the evening's talk, he with his wife gave me the history of their daughter's teeth, a young and most interesting lady not yet hardly grown. It seems from their statement, that the teeth of this girl began to loosen from absorption of the alveolus and recession of the gums when she was only thirteen; and she had soon lost several in this way; while all the others above and below were so loosened, as to promise soon to follow the same road. Indeed they were in such a loose state that the parents consulted a dentist with a view to having them all extracted, thinking it as well to anticipate what seemed an inevitable fate. Before fully deciding on this step, however, (and I should say that various attempts had been made by different dentists to arrest the disease without avail,) a practitioner of medicine of this city of Richmond was consulted, and he gave it as his opinion that the case was one of mercurial poisoning, occurring twelve or more years previously, when the patient was then a small child. Her parents are very positive in their statement that she was never salivated, though admitting that considerable mercury was administered. The M. D., they reported as saying that it would take about ten years or so for this mercurial disease to culminate, which would bring it about to the time from when the mercury was given, to its alleged local expression three years ago. I confess my surprise at this diagnosis, coming even as it does from an unusually acute gentleman, for I had not supposed such local expression of mercury possible after so long lying dormant. But if the diagnosis was startling, the prognosis was still more so, for he advised the patient by no means to have the remaining teeth removed, as in case she did so the disease now locally expressed, might and probably would, be transfered to some internal organ with grave constitutional effects. I have no comment to make upon this, to me, new and very extraordinary diagnosis and prognosis of a disease which we as dentists have been taught to look upon not of mercurial origin, and as involving no such grave consequences as have been here fore-shadowed, and only have to say that if it be true, the sooner we stop the administration of mercury to children the better.

Dr. Thackston. I do not think this disease is of mercurial origin. It is barely possible that the mercury may have tended to induce it, but not to produce it, or to involve the grave consequences here stated.

The Association now proceeded to the election of officers for the ensuing year, with the following result:

President.—Dr. W. W. H. Thackston, of Farmville, Va.

First Vice President.—Dr. J. Hall Moore, of Richmond,

Second Vice President.—Dr. S. H. Henkel, of Staunton, Va.

Third Vice President.—Dr. Geo. B. Steele, of Richmond.

Treasurer.—Dr. James F. Thompson, of Fredericksburg, Va.

Recorning Secretary.—Dr. Geo. F. Keesee, of Richmond, Va.

Corresponding Secretary.—Dr. Jno. Scribner, of Charlottesville, Va.

Reporting Secretary.—Dr. H. M. Grant of Abington, Va.

Executive Committee.—Drs. L. M. Cowardin and Jeter of Richmond, and Dr. Frank Harris, of Harrisonburg, Va.

The President announced that the business of the Association had been accomplished, and it was now ready to adjourn.

Prof. Hodgkin.—Before this Association adjourns, Mr. President, may I be allowed to say a few words more to you, whom I feel to be my brethren, not only in the fact that we are laboring in a common cause, but because I too claim my Virginia birth-right. Were I a native of any other state, I should feel, as I part from you to-night, that I would not be doing my duty if I failed to say that I thank you for your cordial reception of me here, for your respectful attention to the views I have presented, and for the kind hospitality of your homes. But, we "who are to the manor born" know that Virginians don't know any better, sir, than to treat their visitors as you have treated me, and so I make no further mention of it, save to say that I appreciate it. I said just now that I too am a Virginian and if I ask you who are so freely with us in this work of healing, your support in the specialty to which I have been called, that of teaching,—I feel sure that I shall not ask vainly. It may seem a slight thing in me to say that I need all the encouragement and moral support I can get from you in whose ranks I so lately stood as a co-worker, but I declare to you that so heavy does the responsibility weigh upon me, that I feel sometimes like running away from it. To feel that the responsibility is on me of training the minds of young men who are to take the place of men like you sir, is no light thing, and I ask the sympathy and cordial support of you my brethren in this work. But may I ask you also one thing more. In sending us students, as you will from time to time, send such as will reflect credit on your judgment in selecting, and care in teaching, and who will honor the institution of which most of you are alumni.

It will interest you all I am sure, to know that there will be a general meeting of the Alumni of the Baltimore College of Dental Surgery, during the commencement week, on the eighth of March next, to which in the name of the Faculty I cordially invite you.

The Association then at 8-30 P. M. adjourned to meet in Richmond, in December, 1876.

